# Impact of the COVID-19 Pandemic on Tobacco Sales and National Smoking Cessation Services in Korea

**DOI:** 10.3390/ijerph19095000

**Published:** 2022-04-20

**Authors:** Jinyoung Kim, Sungkyu Lee

**Affiliations:** 1 Korea Center for Tobacco Control Research and Education, Seoul 06136, Korea; jy9651@daum.net; 2 Department of Epidemiology and Health Promotion, Institute for Health Promotion, Graduate School of Public Health, Yonsei University, Seoul 03722, Korea

**Keywords:** tobacco, smoking, tobacco sale, smoking cessation, COVID-19, South Korea

## Abstract

This study aimed to describe the effect of the COVID-19 pandemic, combined with the Korean government’s response to the pandemic on tobacco consumption and national smoking cessation services among the Korean population. We obtained tobacco sale data from the Ministry of Finance and analysed the data on smokers’ visits to national smoking cessation clinics during the pandemic from a member of the National Assembly. We also conducted an online search to understand smokers’ thoughts about their tobacco use during the pandemic. We found that after the emergence of COVID-19 in 2020, the sale of conventional cigarettes increased from 3063.70 to 3209.70 million packs (4.77%). The number of smokers who visited clinics sharply decreased in the first half of 2020. The six-month quit rate decreased from 38.5% in 2017 to 22.3% in early 2020. We also found that smokers increased their consumption and began to switch from conventional cigarettes to heated tobacco products. The COVID-19 pandemic has threatened tobacco control policies and programs in Korea in the last two years; however, based on our experience during this period and considering the WHO recommendation, we should sustain and reinforce tobacco control policies and national smoking cessation services today and in the future.

## 1. Introduction

During the first half of 2020, more than one million smokers in the United Kingdom (UK) successfully quit smoking because of the COVID-19 pandemic [1]. The pandemic provided an unprecedented opportunity for UK smokers to quit smoking. Before the COVID-19 pandemic, in 2019, 14.1% of people aged 19 years and above in the UK said they smoked cigarettes; however, after the pandemic, it dropped to 12.3% in 2020 [2]. In India and Italy, the sales of tobacco products were banned when the countries went into lockdown to prevent the spread of COVID-19, because the virus could reportedly spread while smoking [3]. In Hong Kong, the number of quitline participants has increased since the COVID-19 outbreak. Many participants (68%) in Hong Kong’s quitline did not realize that tobacco use potentially increased their risk of developing and spreading COVID-19; however, 43% agreed that the pandemic motivated their intention to quit, while 83% changed their smoking behavior during the pandemic [4]. Given the possible association between smoking and COVID-19 [5], the pandemic has provided an opportunity to reinforce tobacco control policies worldwide. The World Health Organization (WHO) emphasized, on the basis of the results of a review of studies convened by the WHO, that smokers are more likely to develop severe COVID-19 than non-smokers [6]. The WHO recommends that smokers take immediate steps to quit by using proven methods, such as quitline and nicotine replacement therapies, and requests all governments to sustain and reinforce tobacco control [7].

In the Republic of Korea (hereafter Korea), President Jae-in Moon raised the first issue related to COVID-19 and smoking. On 2 February 2020, a Korean evacuation plane returned to Seoul from Wuhan, China, and 360 evacuated Korean citizens were isolated at two different public facilities in Asan and Jincheon, the country’s central region. A week later, President Moon visited Jincheon to comfort the evacuees. During his visit, the Minister of Public Administration and Security, who served as the Minister of Health and Welfare in 2013, reported that smokers vehemently protested against not being allowed to smoke in the rooms of the smoke-free buildings, as well as not being allowed to go out of their rooms to smoke. President Moon addressed this issue by emphasizing the need to do the right thing and offered support to the smokers to quit smoking during such a difficult time [8].

The message from President Moon seemed to have provided a good opportunity to sustain tobacco control policies and comprehensive smoking cessation services during the pandemic. However, unlike President Moon’s thoughts about smoking during the pandemic, the Ministry of Health and Welfare (MOHW) frequently sent official documents to 254 public health centers that provided smoking cessation services all over the country and other national smoking cessation facilities, suggesting that all smoking cessation services be stopped, in order to focus on fighting the spread of COVID-19 [9].

Nevertheless, the recommendation from the WHO was to sustain and reinforce tobacco control policies and comprehensive smoking cessation services during the COVID-19 pandemic, in order to protect smokers, who are more vulnerable to COVID-19 [7]. On the basis of the evidence representing the association between COVID-19 and smoking, the MOHW announced that it included smokers in the populations most vulnerable to COVID-19 in the early stage of the pandemic [10]. However, the MOHW did not sustain smoking cessation services or help smokers quit during the pandemic. It is worth studying the impact of these actions by the MOHW on tobacco use in the Korean population during the pandemic. Hence, this study aimed to describe the effect of the COVID-19 pandemic, combined with the Korean government’s response to the pandemic on tobacco use among the Korean population, especially on tobacco consumption and national smoking cessation services.

## 2. Materials and Methods

The ideal approach to examine the impact of the COVID-19 pandemic and MOHW’s response to the pandemic on tobacco sales or consumption and smoking behavior in Korea is to observe the changes in smoking prevalence before and after the pandemic. However, given that the data on smoking prevalence in 2020 had not yet been released, as of November 2021, we tried to obtain data from three different data sources and triangulate the information to achieve the research aim. Firstly, we considered tobacco sales data as an alternative source to determine the impact of COVID-19. Specifically, we visited the official website of the Ministry of Finance and obtained quarterly tobacco sales data for the 10-year period of 2011–2020, which was the longitudinal quantitative data. The data are publicly available at https://www.moef.go.kr/ (accessed on 1 April 2020). The tobacco sales data included the sales of conventional cigarettes and heated tobacco products (HTPs, since 2017). HTPs are treated as tobacco products, based on the tobacco definition in Tobacco Business Act [11]; thus, they are tightly regulated as conventional cigarettes in Korea.

Secondly, in order to obtain the data on the number of smokers who visited national smoking cessation clinics in public health centers during the pandemic, we worked with Mr. Young-in Koh, a member of the National Assembly. The MOHW has a data collecting system for the national smoking cessation services. All the smoking cessation counselors who work for the smoking cessation clinic in public health centers should upload their records during the smoking cessation counselling. This data has been used to evaluate the national smoking cessation service; thus, they are official and accurate. This data has not been regularly opened to the public, and the MOHW normally releases this data to the public for their purpose or upon the request from news reporters or any members of the National Assembly. In these circumstances, we had contacted a member of the National Assembly. There is a particular system in which the National Assembly requests any kind of information and data from the government. Working with a member of the National Assembly, Mr. Koh’s office, we requested to the MOHW the number of visitors in smoking cessation clinics in public health centers between 2017 and 2020, and the six-month success rate of quitting, all of which can be important criteria for smoking cessation evaluation. Before the COVID-19 pandemic, the six-month success rate of quitting in smoking cessation clinics in public health centers was defined as carbon monoxide (CO) verified abstinence or self-reported abstinence from all tobacco products. This was based on point-prevalent abstinence. However, after the pandemic, the public health centers collected self-reported abstinence. In the context of the pandemic, the smoking cessation clinics were not able to conduct CO monitoring.

Thirdly, we conducted an broad online search on the biggest internet portal in Korea, Naver (www.naver.com), by using keywords such as “COVID-19 and smoking”, as well as on Internet blogs that had any communication about the COVID-19 and smoking, to understand smokers’ thoughts about smoking and their tobacco use during the COVID-19 pandemic. Throughout the first week of October 2021, we searched news articles that were publicly released from April 2020 to September 2021, smokers’ comments from the news articles that we selected, and Internet blogs related to our research topics. We first reviewed the titles of the news articles and excluded duplicated articles, as well as the articles with titles that were not related to our research topics. Afterwards, we read the news articles and looked through the public comments located under the new articles. Anyone can add their comment to most new articles released on the Internet, and it is very common culture to attach their thoughts and opinion to new articles in Korea. This is a popular method to understand public opinion in Korea. This method was not a systematic and comprehensive search, and we tried to briefly collect public opinion related to the COVID-19 pandemic and smoking in Korea. Without any funding source, we were not able to conduct any former survey, which might be a better way to collect public views; thus, we decided to identify smokers’ views, related to the pandemic and their smoking behavior, with this method.

## 3. Results

### 3.1. Sales of Conventional Cigarettes and Heated Tobacco Products

**Figure 1:**
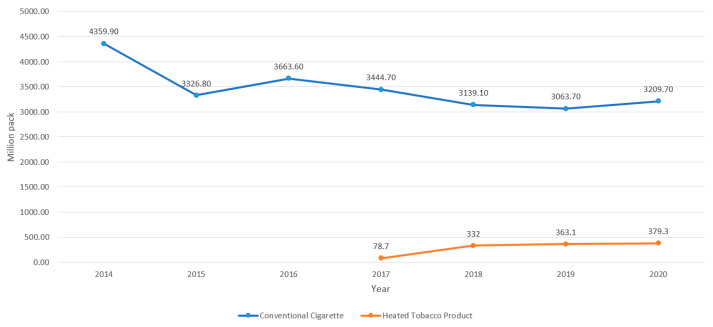
Sales of conventional cigarettes and heated tobacco products in Korea, 2014–2020 (source: Ministry of Finance).

Figure 1 shows the sales of conventional cigarettes and HTPs in Korea from 2014 to 2020. The sales of conventional cigarettes decreased from 3663.60 million packs in 2016 to 3063.70 million packs in 2019; however, after the emergence of COVID-19 in 2020, it increased to 3209.70 million packs (4.77%). After an 80% tax increase on conventional cigarettes in 2015, the sale of conventional cigarettes decreased dramatically, and this continued until 2019, although there was rebound in the sale of conventional cigarettes between 2015 and 2016. HTPs were introduced in the Korean market in 2017, and the sale of HTPs increased rapidly from 78.7 million packs in 2017 to 332.0 million packs in 2018; the sales consistently increased in 2020.

### 3.2. Participants and Smoking Cessation Success Rates in National Smoking Cessation Services

**Figure 2:**
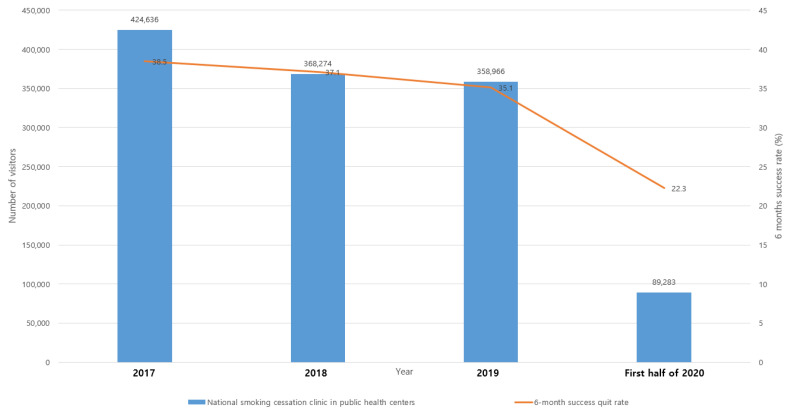
Numbers of smokers who visited national smoking cessation clinics in public health centers from 2017 to the first half of 2020 (source: Ministry of Health and Welfare).

Figure 2 shows the number of participants and smoking cessation success rates for national smoking cessation services from 2017 to the first half of 2020. The number of smokers who visited national smoking cessation clinics in public health centers sharply decreased in the first half of 2020. In 2017, 424,636 smokers participated in smoking cessation clinics; however, in the first half of 2020, only 89,283 smokers visited clinics to quit smoking. In addition, the six-month success rate decreased from 38.5% in 2017 to 22.3% in the first half of 2020. Smokers who wanted to quit during the COVID-19 pandemic did not seek smoking cessation services, because such services were halted by the government. This resulted in a sharp decrease in the number of participants in smoking cessation services. In addition, the drop in the smoking cessation success rate from 38.5% in 2017 to 22.3% in early 2020 can be related to the COVID-19 pandemic. Since the pandemic, national smoking cessation services have only provided support by phone or through text messages, instead of conducting face-to-face consultations.

### 3.3. Smoking Behavior Change during the COVID-19 Pandemic

We searched for relevant references on the biggest Internet portal in Korea, Naver, during the first week of September 2021, to examine smokers’ thoughts about smoking and COVID-19 and their smoking behavior change during the pandemic. We found 480 news articles with the keyword, “COVID-19 and smoking” and firstly excluded duplicated articles that had similar article titles. Then, we read the titles and also excluded the articles that were not related to our topics. With this process, we finally selected 25 news articles for the analysis. The articles mainly reported international research outcomes that informed smokers were more valuable to COVID-19 [12,13] and possible COVID-19 infection in smoking areas where smokers gathered for smoking [14,15]. Although there were a few public comments related to smoking behavior change during the COVID-19 pandemic, we found that smokers who had heard about the relationship between smoking and the severity of COVID-19 wanted to quit smoking and tried to visit smoking cessation clinics in public health centers [16]. We also found that smokers hesitated to enter smoking rooms or areas where they smoked every day during the COVID-19 pandemic. They worried that there were too many people in these areas and felt anxious about taking off their masks to smoke [17]. A comment we have found in the Internet blog said due to the COVID-19 pandemic, he or she smoked indoors without any concern of infection of COVID-19 [18]. With the possible spread of COVID-19, smokers tended to smoke indoors, so as to avoid smoking in public areas.

We also found that since the emergence of COVID-19, Korean smokers have become more likely to increase their consumption. They have more time to smoke while working at home. Before the pandemic, they had to be mindful of other colleagues when they stepped out to smoke, but during the pandemic, they could smoke whenever they wanted to because they worked from home [19]. Consequently, many smokers may switch from conventional cigarettes to HTPs to reduce the smell of tobacco, which is a serious concern in indoor smoking. With this transition, new tobacco products, especially HTPs, have become popular among Korean smokers, and there was consistent increase of the HTP sales in 2020 (during the pandemic). Smokers use HTPs for indoor smoking and conventional cigarettes for outdoor smoking. Taking advantage of this change, the tobacco industry has been promoting HTPs, claiming that their brands have minimal odor and are suitable for indoor use. 

## 4. Discussion

The COVID-19 pandemic has affected the increase sales of both conventional cigarettes and HTP in Korea. In the absence of the COVID-19 pandemic, the sale of conventional cigarettes would have continually decreased, because smokers found it difficult to get an opportunity to smoke because of social norms that denormalized smoking. This increase in tobacco sales can be interpreted in two ways: Korean smokers might have smoked more during the pandemic, more people smoked, or fewer smokers tried to quit during the pandemic. Either way, the pandemic may have harmed tobacco control and public health in Korea.

The MOHW released their mass media campaign with the message, “Smokers should quit during the COVID-19 pandemic” from April to May 2021 [20], because smoking was associated with increased severity of COVID-19. The campaign was widespread, and many smokers were exposed to it. However, smokers experienced difficulty in availing smoking cessation services. With the fight against COVID-19 as the top priority, the MOHW stopped public health centers around the country from providing national smoking cessation services. This may have led to fewer smokers attempting to quit or successfully quitting. Smokers who had heard about the relationship between smoking and severity of COVID-19 wanted to quit smoking and tried to visit smoking cessation clinics in public health centers. Unfortunately, the relevant services had been stopped, because the staff in public health centers changed their roles to COVID-19-related jobs. Unlike in Korea, in Hong Kong, the number of quitline participants has increased since the COVID-19 outbreak. Almost half of the participants said that the pandemic motivated their intention to quit, while 83% changed their smoking behavior during the pandemic [4].

The COVID-19 pandemic may serve as a potential tool to motivate smokers to quit smoking. They were concerned about their smoking behavior. For example, Korean smokers worried that there were too many smokers in smoking rooms or areas where they smoked every day, and they felt anxious about taking off their masks to smoke. The pandemic has, thus, presented a good opportunity to encourage smokers to participate in quitting. Therefore, we should prioritize sustaining and reinforcing smoking cessation services and tobacco control policies during the COVID-19 pandemic or when facing other types of infectious respiratory diseases in the future.

The tobacco industry has been more active in using the COVID-19 pandemic for marketing HTPs. The industry claims that HTPs do not create any tobacco smoke or odor and they generate aerosols that are much less harmful than conventional cigarette smoke [21]. Given the situation wherein smokers are spending more time at home during the COVID-19 pandemic, they can be more interested in using new tobacco products, following the industry’s claims. However, it is important to mention that this type of smoking behavior causes dual use of conventional cigarettes and HTPs [22]. Smokers smoke conventional cigarettes when they are outside but smoke HTPs when they are at home or indoors. The government should closely monitor the tobacco industry’s marketing activities and educate smokers about the dangers of using HTPs use. Related to the tobacco industry’s activities during the pandemic, the ninth session of the Conference of the Parties (COP) to the WHO Framework Convention on Tobacco Control (FCTC), which was held on 8–13 November 2021 in Geneva, Switzerland, adopted a declaration in the WHO FCTC and recovery from the COVID-19 pandemic. The declaration calls on 182 FCTC parties to take appropriate measures to prevent tobacco industry interference and involvement in COVID-19-related public health policies and actions in accordance with Article 5.3 of the WHO FCTC and its guidelines for implementation [23].

There were strengths and limitations in this study. The use of longitudinal data from large, routinely collected datasets for the analysis of tobacco sales was a strength, however, there were some limitations, including the possibility of confounding variables impacting the tobacco sales, and the lack of currently available data on smoking prevalence in Korea. It was unclear how reliably sales of tobacco reflect real tobacco consumption and whether there was an illicit trade of tobacco. The data we used on the numbers of smokers visiting national smoking cessation clinics did not seem to be comparable, as data from each of the preceding three years were compared with data from the first half of 2020, and it might be difficult for the reader to meaningfully interpret this. Unfortunately, the MOHW did not provide the raw data on the numbers of smokers visiting national smoking cessation clinics to the member’s office of the National Assembly. However, there is a seasonal effect, whereby Korean smokers are more likely to visit smoking cessation clinics in the first half of the year than the other; thus, we believe that there was a huge reduction in the numbers of smokers visiting national smoking cessation clinics during the first half of 2020. The findings on smoking behavior change during the pandemic (from the Internet) did not represent the majority views of Korean smokers; thus, the finding should be carefully interpreted. In addition, as the findings were collected in the cross-sectional analysis, there was a limitation to infer real changes to smoking patterns at the population level.

## 5. Conclusions

This study aimed to describe the effect of the COVID-19 pandemic, combined with the Korean government’s response to the pandemic on tobacco consumption and national smoking cessation services among the Korean population. We found that, after the COVID-19 pandemic, the tobacco sales increased and the number of smokers who visited smoking cessation clinics sharply decreased in the first half of 2020. We also found that smokers increased their tobacco consumption and began to switch from conventional cigarettes to HTPs. Consistent with Article 14 of the WHO FCTC, the ninth session of the COP to the WHO FCTC aimed to explore health system adaptations to support alternative service delivery options, such as e-health and telemedicine consultations, for tobacco dependence and cessation services [23]. Similar to the WHO’s recommendation, there were Internet- and telephone-based smoking cessation services for smokers who wanted to quit smoking during the pandemic in Korea. If they were effective during the pandemic, in the Korean context, they can be useful and valuable and, thus, need to be expanded further in the future. The COVID-19 pandemic has threatened tobacco control policies and programs in Korea in the last two years; however, based on our experience during this period and considering the COP declaration, we should sustain and reinforce tobacco control policies and national smoking cessation services today and in the future.

## Data Availability

The data about tobacco sales in the Republic of Korea are publicly available at https://www.moef.go.kr/ (accessed on 1 April 2020).
